# Scaling Early Parenting Interventions: A Qualitative Investigation of Factors that Enable Successful Scaling of Early Parenting Interventions for Sustainable Implementation and Impact

**DOI:** 10.1007/s10488-026-01500-2

**Published:** 2026-04-06

**Authors:** Jane Kohlhoff, Martina Donkers, Sara Cibralic

**Affiliations:** 1 School of Clinical Medicine, Discipline of Psychiatry and Mental Health, UNSW Sydney, Sydney, Australia; 2 Karitane, Carramar, Australia

**Keywords:** Early parenting interventions, Program scale-up, Implementation science, Stakeholder engagement, Mental health services

## Abstract

Recent decades have seen emergence of numerous evidence-based early parenting interventions. To make an impact at a population level, they need to be delivered at scale. This study aimed to identify factors that enable successful scaling of early parenting interventions for sustainable implementation and impact. Participants were 22 individuals from the United States of America, Australia, Korea and the Netherlands, all who had experience in the development, implementation dissemination and/or scaling of early parenting interventions. Participants completed in-depth interviews about their experiences or observations of scaling early parenting intervention, and enablers and barriers to successful scaling. Transcripts were analysed using thematic analysis. Results revealed **s**ix key themes: (1) adopting a business mindset, including sustainable funding and governance models; (2) securing multi-level stakeholder buy-in, from clinicians to policymakers; (3) implementing flexible, context-sensitive models that support fidelity and adaptation; (4) ensuring intervention quality, including evidence-based design and cultural relevance; (5) assembling multidisciplinary teams with the necessary expertise and leadership; and (6) time and planning. Results suggest that scaling early parenting interventions is a dynamic, non-linear process that can take considerable time and planning. Scaled interventions need to be evidence-based and culturally relevant, but there also needs be a sound business model, widespread stakeholder involvement, and strong leadership. Sensitivity and flexibility to meet the needs of local contexts are also vital. Taken together, this study offers actionable insights for policymakers, funders, and practitioners seeking to expand the reach and impact of early parenting interventions within diverse service systems.

Converging evidence highlights the foundational role of the early caregiving environment in shaping children’s social, emotional, and psychological development (Bourne et al., 2022; Vermeer et al., 2016). Early parenting programs present a valuable opportunity to support early caregiving through strengthening parenting skills, and improving parenting knowledge, attitudes, beliefs and feelings during the critical developmental window of early childhood (defined here as 0–6 years) (Butler et al., 2020; Prime et al., 2023; World Health Organization, 2024). A wide variety of early parenting programs are available. Some are grounded in attachment theory, emphasising the importance of secure parent-child relationships, e.g., Child-Parent Psychotherapy (CPP; Lieberman et al., 2006), Attachment and Biobehavioral Catch-Up (ABC; Dozier & Bernard, 2017) and Circle of Security (COS; Powell et al., 2014). Others draw on social learning theory, focusing on behaviour modelling, reinforcement, and skill-building, e.g., Parent-Child Interaction Therapy (PCIT; Eyberg, 1988) and Triple P – Positive Parenting Program (Sanders et al., 2000). There is also variety in the modality in which early parenting programs are delivered, ranging from group-based psychoeducation to individualised coaching or counselling (Prime et al., 2023; Wyatt Kaminski et al., 2008). Programs are also differ in their placement along the prevention–intervention continuum, ranging from universal programs aiming to promote positive parenting across the population, to targeted interventions addressing specific risk factors such as trauma exposure or behavioural difficulties (Sanders & Turner, 2018; Toumbourou et al., 2024).

The evidence base for many early parenting programs is robust, with numerous randomised controlled trials demonstrating improvements in parenting practices, child behaviour, and family functioning (Branco et al., 2022; Doyle et al., 2023; Jeong et al., 2021). Many of these research trials, however, have been conducted in academic settings with restrictive inclusion/exclusion criteria and high levels of clinical training and fidelity, making it difficult to ascertain effectiveness in real-world settings (Hickey et al., 2021). In recent years, significant progress has been made in this area with the emergence of ‘Implementation Science’ as a discipline (Bauer & Kirchner, 2020). Various implementation science frameworks have been developed to guide the implementation of health innovations (Aarons et al., 2011; Damschroder et al., 2009; Holtrop et al., 2021), many of which have been used in hybrid effectiveness-implementation evaluation research designs (Curran et al., 2012). With regards to early parenting programs, there are now many documented examples where Implementation Science principles have been used to support the effective implementation of early parenting programs in real-world contexts, particularly in low- and middle-income countries where contextual demands are typically very different to those in which original research trials were conducted (Lansford et al., 2022). Emerging evidence from qualitative studies has further highlighted specific facilitators and barriers (at system, provider, program, organisation and client levels) to implementing parenting programs in real-world contexts (Cooper et al., 2022).

In addition to implementing early parenting programs effectively, it is increasingly acknowledged that in order to make an impact at a population level, programs also need to be delivered at ‘scale’ (World Health Organization, 2024). In healthcare, ‘scaling’ is understood as a deliberate and structured process aimed at broadening the reach and impact of proven health innovations to generate population-level benefits and drive lasting changes in policy and service delivery (World Health Organisation, 2009; World Health Organization, 2010). Fixsen et al. (2017) further describe scaling as the extent to which an innovation is successfully adopted across its intended population.

The literature has proposed a differentiation between different scaling ‘types’ or ‘directions’, which has provided useful nuance to the field and clarity for individuals/groups who are doing this work. The ExpandNet framework (World Health Organization, 2010), for example, identifies four distinct scaling pathways: 1) Vertical scaling up, which involves embedding innovations into institutional systems through changes in policy, governance, regulation, and financing at national or sub-national levels; 2) Horizontal scaling up, referring to the replication or geographic spread of an innovation to reach wider or more diverse populations; 3) Diversification (also known as functional scaling), which integrates new innovations into existing scale-up efforts; and 4) Spontaneous scaling up, where innovations diffuse organically without formal implementation strategies. Complementing this, Moore et al. (2015) propose three strategic directions for scaling social innovations: 1) Scaling out, which focuses on replicating effective models across communities to increase reach; 2) Scaling up, which targets systemic change through shifts in institutional policies and governance; and 3) Scaling deep, which seeks to transform cultural norms, values, and relationships to embed lasting change. Britto and colleagues bring a perspective of particular relevance to the field of parenting, conceptualising scaling as both expanding programs to enable access by larger populations (i.e., “small to bigger”), and leveraging existing at-scale systems and supplementing or enhancing them to include focus on parenting (i.e., “big to better”) (Britto et al., 2022).

With the increased focus in recent years on implementation and scaling of early parenting programs, there has also been a proliferation of guidelines and resources to support this work. Various frameworks have been developed to guide the scale-up of healthcare innovations more generally (Milat et al., 2014; World Health Organization, 2024). Peak bodies have also developed standards for efficacy and effectiveness, recommending that research about scale-up processes be integrated into all stages of the implementation and scaling process (Gottfredson et al., 2015). In relation to parenting programs specifically, the World Health Organization has recently published a guide to “designing, implementing, evaluating, and scaling up parenting interventions” (World Health Organization, 2024). These guidelines articulate three clear phases to be undertaken when scaling parenting programs, namely laying the groundwork for implementation (phase 1), implementing the intervention (phase 2), and engaging in learning and sustainability (phase 3), and provide step-by-step guidance around key tasks.

Taken together, it is clear that there is now increasing acknowledgement of the importance of implementation and scaling of early parenting programs and growth in the guidelines and resources to support implementation and scaling. A number of published qualitative studies have identified facilitators and barriers to the implementation of parenting programs in real-world contexts (Cooper et al., 2022) but the focus of much of this work has been on implementation rather than scaling. As a result, there is a gap in understanding about facilitators and barriers to the successful scaling of early parenting interventions, and a need for research that investigates the experiences and perspectives of groups or individuals who have been involved in real-world scaling efforts. To address this gap, the current study sought to explore the lived experience of individuals who had been involved or closely observed the scale-up of an early parenting intervention. The specific aim was to identify factors that enabled successful scaling of early parenting interventions and the challenges that must be overcome to ensure programs remain effective and sustainable in diverse real-world settings.

## Method

### Overview of Methodological Approach

This study sought to explore the experiences of individuals who had direct experience with scaling early parenting interventions. Data was collected through videocall interviews, and analysed using an inductive thematic analysis approach, within a phenomenological and essentialist-realist theoretical framework (Braun & Clarke, 2006, 2022). This allowed for themes to be identified from the data based on the experiences of participants and the meanings that they attributed to them, without the imposition of pre-existing or pre-determined theories or categories. In this way, data was evaluated on a semantic level, with themes identified within the surface meaning of data rather than from inferences about meaning beyond what was said (Braun & Clarke, 2006, 2022).

### Participants

Participants were 22 ‘knowledge experts’ who had gained their knowledge through direct involvement in scaling an early parenting intervention. Most participants were interviewed individually (*n* = 20; 90.9%), with the remaining two interviewed as a pair (speaking about the same scaling effort). Twelve participants were recruited following identification through a scoping review (undertaken by the lead author; Unpublished). Snowball sampling was then used to identify and recruit an additional 10 participants. Participants were recruited until data saturation was reached, with no new emerging themes.

### Procedure

All interviews were conducted between February 2025 and April 2025, by the first study author (clinical psychologist and academic researcher with over 20 years of experience working in the field of perinatal, infant, and early childhood mental health). Interviews were conducted and recorded via video conference (MS Teams or Zoom) and were guided by a semi-structured interview schedule format comprising broad, open-ended questions, developed to explore the study aims and allow for consistent questioning across the interviews while also allowing for exploration of topics arising during the interview. Example questions included: “Tell me about your experiences of scaling [name of intervention] in [name of place/region]”, “what things helped the scaling effort to succeed?”, and “what were some of the challenges that you/your team faced?” Interviews were 45–60 min in length and were transcribed verbatim using Otter AI, with identifying information removed from the transcripts.

Coding and analysis were undertaken from June 2025 – August 2025 by the three study authors (the second and third study authors contributed to data coding and analysis but did not complete any of the participant interviews) using a collaborative, interactive, and iterative coding approach that allowed for the sharing of divergent viewpoints on data analysis and theory development (Birks et al., 2019). Each of the coders brought unique perspectives to the data: two were clinical psychologists and research academics with professional expertise in early parenting interventions and lived experience of parenting young children (JK and SC); the third coder (MD) was an evaluation specialist with expertise and experience in qualitative methodology, program implementation and systems change. Each coder coded 50% of the transcripts (50% of the transcripts were double coded), across five sequential steps. First, co-authors read the transcripts independently to familiarise themselves with the data. Second, each coder examined the transcripts line by line, applying initial codes to each line. Third, each individual coder reviewed their initial set of codes and developed overarching categories (themes and sub-themes) that subsumed most of their initial codes. Fourth, the co-authors met to share and discuss their individual codes and categories, and through a process of group discussion, refine the categories, identify themes and subthemes, and develop a coding scheme/code book that articulated these themes and subthemes. If coding disagreements arose, they were used as a gateway to reflection and discussion, which aided the reflexive process. Fifth, all transcripts were re-coded according to the coding scheme/code book. This served both as a second check of the validity of the coding scheme and as an opportunity to identify quotes that best illustrated the identified themes and subthemes. The extracts that appear in this paper were chosen on the basis that they provide the clearest descriptions of themes relevant to the current study. The transcribed interviews have been edited so repeated words and phrases, and irrelevant words such as “um” and “like” have been removed, and irrelevant sentences have been replaced with ellipses ( …). Data coding was conducted using the computer software, NVivo.

### Ethics approval

The study was approved by the University of New South Wales human research ethics committee (approval number:7770 ). All participants provided written, informed consent.

## Results

**Fig. 1:**
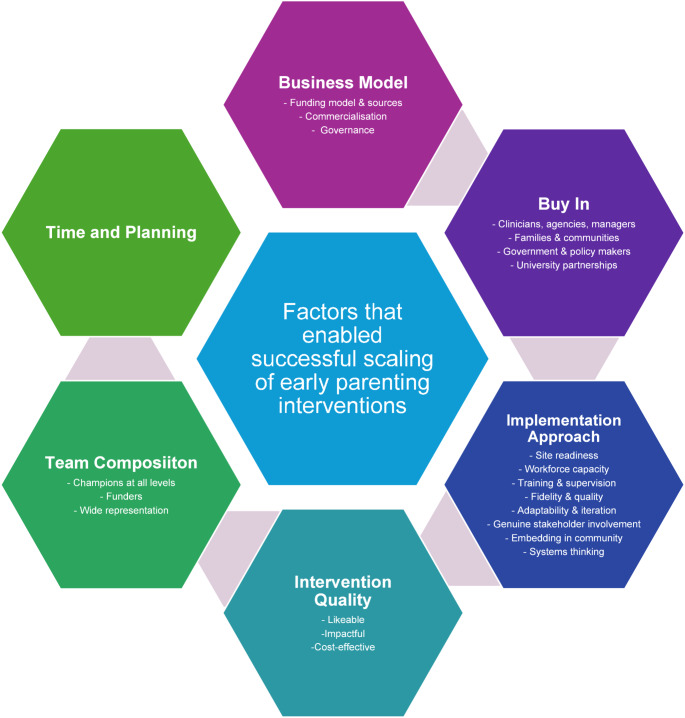
Themes and subthemes

Participant characteristics are shown in Table 1. As shown, the majority of the 22 participants were from the United States of America (USA; *n* = 14, 64%), followed by Australia (*n* = 6, 27%), Korea (*n* = 1, 4.5%), and the Netherlands (*n* = 1, 4.5%). Around 27% were male and 77% female. Seventeen of the participants were directly involved in scaling an early parenting intervention, and of these, 5 were developers of the program; four participants were involved in policy or systems work and were able to provide insight based on interactions and observations of scaling efforts. Participants spoke about a range of scaled early parenting programs including Parent-Child Interaction Therapy (*n* = 5), ABC Biobehavioral Feedback (*n* = 1), Circle of Security (*n* = 1), Child Parent Psychotherapy (CPP; *n* = 1), Family Check Up (*n* = 2), Managing and Adapting Practice program (MAP; *n* = 1), Maternal Early Childhood Sustained Home-visiting (MESCH; *n* = 1), nurse home visiting in Korea (*n* = 1), Restacking the Odds (*n* = 1), smalltalk (*n* = 1), Triple P (*n* = 1), and Video-feedback intervention to promote positive parenting (VIPP; *n* = 1). Some of the participants discussed efforts to scale multiple interventions (e.g., PCIT, ABC, CPP in a USA state) and others were not aligned to any one intervention but spoke more broadly of knowledge gained from position of state or leadership in the field of perinatal and infant mental health (*n* = 4). Some participants were the original program developer (*n* = 6; 26%), others were not a program developer but had been involved in scaling the program across a specified region (*n* = 12; 52%), and others had knowledge of the scaling effort due to a role as policy maker or contribution to systems-change at a broader level (*n* = 4; 17%). Data analysis yielded six major themes and 21 subthemes: (1) Business Mindset (funding model and sources; commercialisation; governance); (2) Buy In (clinicians, agencies, managers; families and communities; government and policy makers; university partnerships); (3) Implementation Approach (site readiness; workforce capacity; training and supervision; fidelity and quality; adaptability and iteration; genuine stakeholder involvement; embedding in community; systems thinking); (4) Intervention Quality (likeable; impactful; cost-effective); (5) Team Composition (champions at all levels; funders; wide representation); (6) Time and Planning (Fig. 1).

**Table 1:** Participant demographics

Demographic variable		*N* (%)
Gender	Male	6 (27.27)
	Female	17 (77.27)
Country of Residence	United States of America	14 (63.64)
	Australia	6 (27.27)
	Korea	1 (4.55)
	Netherlands	1 (4.55)
Role in Scaling Initiative	Program developer and scaler	5 (22.73)
	Scaler	12 (54.55)
	Policy/systems	4 (18.18)

### Theme 1: Business Model

Participants noted that for an early parenting intervention to be successfully scaled, there needs to be a business model that is realistic and functional, and that considers funding and governance arrangements.

#### Funding

During both the development of new models and scaling, there should be a proactive consideration of funding models [*“Without the right financial model behind the work*,* you can’t do the work.” – participant 4]*. Importantly, funding arrangements must be supportive of workforce stability. Models that rely on clinicians being contracted and paid only for services when clients attend sessions (e.g., as is the often the case in the US, through Medicaid) are not viable because trained clinicians often seek positions with financial security. To support long-term attraction and retention of clinicians, careful consideration should also be given to clinician salary rates, ensuring that clinicians are appropriately renumerated for the services they deliver. The costs that agencies bear to train staff (i.e., hours off clinical service provision to attend training and supervision) also need to be considered, and if possible, agencies should be reimbursed for clinician training/supervision time *[“The agencies are saying we’re losing money by getting our folks trained. That’s a big barrier” - participant 1].*

There must also be a clear plan within the business model for how the scale-up will be funded, whether that be from philanthropic doners, government grants, or a payment model. Philanthropic funding can play a valuable role in the early stages of innovation and scaling, supporting catalysation of new ideas, pilot testing, and specific program specific equipment costs, paving a pathway towards government investment for long-term sustainability. *[“In each smaller grant*,* I would test something out that led to the…larger federal grant*,* which was a $3.3 million grant to implement across the state” - participant 4].* To be effective, however, there must be alignment between the strategic priorities and values of the scaled intervention and the philanthropic organisation.

Securing government funding to support scaling and sustainability of early parenting programs is often essential. This can include one-off funding grants to support workforce training and development [“*We had a five-year federal grant that was a workforce development grant*,* and through that*,* we reach*,* we partnered with five rural counties” - participant 3*], government-driven scale-up across large geographical areas *[“The central government has started to expand the program. Right now*,* the central government program has been expanded to 67 districts across the country” - participant 17]* or contractual agreements with government to enable dissemination at a population level [*“The major commitment from government is through the parent education and support program*,* through the [department of health]” - participant 8*]. Scale-up is supported when programs become part of government-funded service infrastructure, e.g., getting the program on the list of ‘billable’ programs through Medicaid/Medicare [“*[intervention] is one of the few*,* very few interventions that are built into the state infrastructure… There is an enhanced rate*,* so providers make more money when they use [intervention] as opposed to standard outpatient practices…I think that that is really important*,* because that’s what sustains it over time”*,* - participant 4*].

Relying on government funding does, however, bring challenges. The uniformity of Medicaid rebates can discourage further professional development and limit the program adoption (e.g., in some US states, Medicaid rates for an intervention remain the same regardless of a provider’s level of training). Changes in government and/or government policy are also a significant threat *[“When the new government came in*,* they just stopped the money…not even just a gradual bit*,* it was just gone” – participant 6].* Even for programs that are deeply integrated with government systems, funding limitations persist and so there is an ongoing need to advocate and work with government to increase funding *[“We were fortunate that we had a lot of government backing and support for the scale up of [intervention]*,* so that now it’s available in all 79 local government areas across [state] to all families who are eligible.…The problem is*,* of course*,* we don’t have the funding or capacity to do everything we’d like to do.” - participant 5].* Lack of policy alignment between federal and state governments can also be an issue, especially when federal decisions influence state-level funding.

#### Commercialisation

Another step in ensuring the long-term viability and sustainability of a scaled intervention is commercialisation. It is common for program developers, who are typically academics or clinicians, to underestimate the importance of adopting a business mindset and commercialisation [“*We always called it the business that didn’t want to be a business” - participant 14].* Many program developers also face significant skill and resource gaps in commercialisation, e.g., the necessary training, time, and incentives to build business models or navigate nonprofit structures. Marketing, legal setup, and infrastructure development are often not recognised as needed; they are also unfamiliar and costly, often requiring personal investment or external funding. As a result, promising programs are often not maintained or disseminated widely. [*“The problem is that most of the scaling models are not based on sound business principles. And so*,* you know*,* many of the innovations that could get out there don’t survive. You know*,* they might start off with a hiss and a roar*,* but they don’t have a business model to sustain it…I think the enablers were to recognise that in order to scale a program*,* to disseminate it*,* you needed a business model. You go broke*,* giving it away*,* and it’s simply not sustainable unless you’ve got a business model underpinning it that will produce a revenue stream that will enable the program to continue to evolve. It’s going to be dead in no time at all.” - participant 8]*

For success, the intervention model and the business model must be well-aligned and designed with scalability in mind. Leveraging any business expertise of team members can be valuable, and initial ‘start-up’ funds are essential. Early funds can be sourced through small philanthropic or business grants, but in many cases, these funds come from program developers’ own personal funds. [*“The formation of it cost $15*,*000 of my own money. So there is some cost up front. You better think you’re going to be able to get that back in profits*,* right?” – participant 13].* It can also be useful to pursue funding streams aimed specifically at supporting commercialisation.

Importantly, ‘commercialisation’ should not be equated with ‘profit-motivated’; businesses can be 'values-based' and viewed as the vehicle through which effective intervention models can have broader reach and impact. [*“You stay ethical*,* you gotta be thoughtful. And if what’s driving you is money*,* good luck to you*,* because that’s not going to work out very well. But if what’s driving you is ‘okay*,* we know this is a helpful tool for folks. How can we continue to deliver it to the many millions of people that may benefit from it?’ We’re just one of those kinds of businesses” – participant 14]*

#### Governance Management Structure

It is important to develop a governance management structure to ensure capacity for large-scale oversight and coordination of the scaling effort. A centralised management approach provides an avenue through which program developers/training organisations can provide agencies and trained practitioners at scaled sites with access to ongoing support and develop professional networks and opportunities for connection (e.g., Communities of Practices), which supports program sustainment and quality/fidelity. *[“When we started*,* there was already quite a lot of activity that was happening in most jurisdictions and particularly in the national frame as well*,* but not connected and not coordinated. So many players who weren’t necessarily talking to each other or who might have done in an ad hoc way*,* the network really aims to bring together more purposefully.” – participant 15]*.

Importantly, centralised governance management structures can also support data collection, reporting and quality monitoring, and funding submissions. In some cases, the centralised body maintains distance from the scaled sites following initial training, viewing their ongoing role as largely to collect data and provide feedback to support quality service provision *[“…it’s very light touch. As long as they continue to do [program name]*,* they need to be providing us with the data*,* but that’s only so it turns around in dashboards to them*,* so they can continue to monitor the quality. And we do a very light sort of oversight - we require sites to have a local steering committee*,* and we attend it once a quarter to just kind of have that light touch base*,* about maintenance of quality and fidelity*,* but otherwise*,* we train trainers then we step out” – participant 6*]. In other cases, a greater degree of control is retained, due to concerns about fidelity. Sometimes this involves mandated requirements for trainers at scaled sites to continue with ongoing supervision with the central team [*“We hold the reins really tight*,* because … I’ve always thought*,* once you let the reins go*,* you can’t get them back…we stay very*,* very tightly connected” – participant 16]*, or funding withdrawals to ensure fidelity *[“I don’t come hard on a program and immediately take their funding away….there’s a whole lot of steps to give them every opportunity to succeed. And then it’s not a punishment*,* it is a learning process*,* like when you can address these things*,* then your money will start flowing again.” – participant 19].*

### Theme 2: Buy-in

For implementation of an early parenting program to be successful, there needs to be ‘buy in’, or in other words support and commitment to the implementation effort, at multiple levels: clinicians and agencies; managers and administrators; families and communities; and government and policy makers.

#### Buy in from Clinicians and Agencies

To gain buy in from clinicians and agencies, it is important to raise awareness of the need for the new program and encourage engagement *[“So we’re at this point right now where we’re working with local agencies…and we’re talking about what infant mental health is….And then we’re saying to the child serving agencies in those communities*,* “Would you like to learn more or have a deeper investment*,* or get your folks trained?’” – participant 23*]. Special efforts to visit sites and create enthusiasm is also another successful strategy *[“We create enthusiasm in the community. We invite all of the agencies… anybody who’s willing to hear our voice….in [new location]*,* we had to create the interest for it*,* because there was no torch bearers out there” – participant 18].* ‘Buy in’ can be further fostered by listening to the perspectives of clinicians and being willing to modify training approaches to suit their needs and preferences *[“So it’s got*,* it’s very flexible*,* and we found that clinicians like it. The dropout rate has gone way down” – participant 1*] and in some cases, using funding to incentivise training and/or use of the intervention [“*Getting an enhanced rate means that they are incentivized to actually provide [intervention name] as opposed to treatment as usual” – participant 4*]. Site visits and regular communication are also key *[“That’s where you develop personal relationships and trust.” – participant 19].*

#### Buy in from Managers and Administrators

There also needs to be buy in from the managers and administrators who supervise the work of the clinicians and oversee the finances/operations of the agencies *[“It’s not enough to have passionate therapists and to do really great training of therapists*,* you’ve really got to train the system*,* and you’ve got to get the administration…. they have to be involved and they have to be just as passionate as the therapist is*,* because it takes a lot of resources to sustain a program over time.” – participant 9].* Focused effort to identify the right person or people to speak with *[“I have to make sure the ‘boots on the ground’ person*,* the office manager*,* knows what’s happening….not just the senior leader*,* not just the administrator*,* but the clinical supervisor for the whole agency. Who’s that…person at that location that’s responsible for making the decisions? I need to talk to them”. – participant 18]*, or deliberate efforts to involve the whole agencies in training, not just the clinicians who will deliver the intervention *[“We want to get the supervisors and the clinicians as a team to come in. It doesn’t work if you just get a clinician trained and the supervisor doesn’t know what you talk about.” – participant 1].*

#### Buy in from Families and Communities

Program developers/scaling teams also need to listen to, and work collaboratively with, communities to identify local needs and select interventions that are most likely to be effective. *[“I think that some of the key pieces of that scaling up were involving the community and key stakeholders in every step of the way*,* and listening to them*,* genuinely listening to them*,* and taking their advice” – participant 4].*

#### Buy in from Government and Policy Makers

Government buy in is also crucial, especially when government funding is needed. Scaling is facilitated when there is alignment with current government policies, initiatives, plans, or frameworks *[“The thing that made the rollout and scale up effective was the support by the government building it in as part of policy and process” – participant 5]*, and often there needs to be active work in collaboration with politicians to change policies to ensure alignment *[“When I think of the things that have helped it to sustain over time*,* it’s the getting it into the infrastructure of our policies and funding.” – participant 4; “Scaling sustainably really hinges on being able to get long-term investment or being able to change policy such that it paves the way for ongoing expenditure” – participant 7].*

Securing buy-in from government can be challenging, with changes in government priorities, and political election cycles presenting significant obstacles. Enabling factors include: opportunistic scaling where government policy/budget conditions are favourable *[“They’ve got a lot of money for early mental health right now” – participant 16];* targeted advocacy *[“We had to persuade the central government” – participant 17]*, education *[“They held an event to educate their state legislature about infant and early childhood mental health.” –– participant 7]*, and deliberate efforts to maintain visibility with government legislator*s [“We talk to our legislators and the people that are in in charge of handing out money… I want them to know all of the work we’re doing*,* because if we disappear*,* the agencies won’t be able to afford the trainings.” – participant 18].* Once there is government buy in, maintaining it requires trust, developed through transparent, consistent communication. *[“We’re meeting on a regular basis with the state leadership…. We have quarterly in person meetings….We have reports that we give them on how many consultations*,* how many trainings*,* how many people attended*,* all of that. So there’s constant flow of information. Nobody’s kept in the dark” – participant 1].*

#### University Partnerships

Partnering with universities can in some cases support intervention developers in grounding their work in evidence-based practice and building credibility with other stakeholders. Universities’ willingness to support scaling efforts benefits those responsible for dissemination by enhancing reach and ensuring they are appropriately compensated for their contributions. There are ongoing challenges with funding any kind of research institute.

### Theme 3: Implementation Model

Successfully scaling early parenting interventions requires application of an implementation model that address site readiness, workforce capacity, sustainable training and supervision, and maintenance of fidelity and quality. Moreover, scaling is most effective when the implementation models also incorporate flexibility for local adaptation, emphasise community embedding, and are ‘systems’ focused.

#### Site Readiness

When scaling interventions, it is important to take care when choosing sites. This includes ensuring that the population the site caters to is compatible with the intervention, that teams are trained in a way that mitigates the issue of staff turnover, and that regular funding is available to continue training new staff members. Successful scaling programs assess site readiness, typically taking into consideration practical set-up issues [“*We go out for a site visit*,* and I make sure they have all the things that they say they have… Do you have the space to do this? Do you have the equipment to do this?… How many kids do you see?” – participant 18*], resources/capacity [*“An interview process…to make sure they have the resources right…leaders…that they actually see families” – participant 12]*, local workforce capacity *[“The scale-up is started in the areas that…are closer to ready in terms of the workforce” – participant 6]* and local infrastructure and support networks *[“What does this particular state look like in terms of funding and champions?”– participant 3]*. Using site readiness assessments to guide decisions around the order in which sites are chosen for implementation is also important *[“Their decision was to start with the regional centers first*,* and that was strategic….because at the regional centres*,* you can have more confidence about the capacity to build the workforce capacity” – participant 6].*

Program implementers often work collaboratively with sites to identify and implement strategies to improve readiness *[“…At the end of the assessment*,* we don’t say*,* “Oh*,* well*,* you can’t participate. You’re not ready”*,* but rather*,* “What do we need to build up to make sure that if you get this training*,* or your staff get this training*,* that you can make good use of it*,* that they can really participate?” – participant 23].* The early phases of learning collaborative models help to enhance site readiness *[*“*We’ve used a learning collaborative approach where you’re doing a lot of administrator training and supervisor training when we go into a newer area*,* with the idea that you’re from the very beginning*,* building up the capacity in an area to be able to be ready for sustainability*,*…when you go in and just do clinician training*,* it’s not enough because the best of clinicians*,* if they don’t get support*,* can’t keep it up. So you have to ready the environment for them to be able to do it*,* and ready the environment with the idea that they’re going to need ongoing support” – participant 4]*.

#### Workforce Capacity

One of the barriers to successful scaling of early parenting programs is poor workforce availability. Workforce shortages are often driven by low salaries and unsustainable work conditions (leading to high staff turnover) and lack of trained specialists, and in rural areas, large travel distances [“*Folks are just really resource constrained and stretched in terms of*,* like*,* how much they can do in their role” – participant 7].* Scaling is most successful, therefore, when there are strategies in place to build and strengthen the capacity of the workforce delivering the parenting program [“*You cannot scale without a workforce development model” – participant 6]*. For some programs, clinician supervision and refresher trainings are provided centrally; in other cases, local clinicians are trained as within-agency trainers (sometimes referred to as a ‘train-the-trainer’ model) [“*We started to grow and develop the workforce*,* trying to get agency trainers at each site*,* and if not at each site*,* with each organization. When we got into our rhythm of about every 18 months doing a trainer training for the people we had trained about 18 months to two years beforehand*,* we started to get a good critical mass of trainers” – participant 18*]. Where workforce shortages arise due to bigger systemic factors (e.g., not enough of a required profession being trained at the university level), larger-scale workforce development models can be enacted using scholarships and incentives to encourage growth in numbers *[“To get an increased number of Child and Family Health Nurses… they’ve developed a workforce model*,* and they’re implementing that now” – participant 6*]. Another way to address workforce issues faced in many areas, particularly in rural areas, has been to loosen the pre-requisites for clinicians to be trained in the model *[“We used to have a high bar*,* like people needed to have a master’s degree or some other clinical expertise. But in the United States*,* essentially*,* pretty rapidly over the past 10 years*,* we’ve moved to a model of mental health delivery here where it’s mostly bachelor’s level people. So there’s really hardly anyone in these community health agencies that has a master’s degree anymore*,* so we pretty much train anyone*,* because that’s the workforce*,*” – participant 13].*Communities of Practice can be used to provide peer-support for clinicians, particularly those working in isolation due to geographical distance [“*In many of the rural regions*,* the teams aren’t as large. Often it’s one person delivering it*,* so they don’t have a team around them….So we do a lot in the community of practices to build up….their connection to each other and encourage their collaboration and support” – participant 5].*

#### Sustainable Models of Training and Supervision

Sustaining an intervention at scaled sites and agencies requires proactive planning. Without a clear sustainment strategy, staff turnover can lead to the loss of capacity to deliver the intervention. Establishing multiple training pathways is essential, with in-house trainers offering a particularly effective solution by enabling ongoing internal training and reducing reliance on external providers. Support mechanisms for clinicians—such as peer support networks and consultation opportunities—also contribute significantly to sustainability by reinforcing practice and maintaining fidelity. Additionally, recent graduates tend to show higher levels of continued engagement with the intervention post-training, suggesting that targeting early-career professionals may be a strategic approach to long-term implementation success *[“It’s now not dependent on me*,* and others have picked up and are running with it and continuing to evolve it” – participant 8].*

#### Mechanisms to Ensure Fidelity and Quality

While manualising interventions can help with scale-up, many early parenting interventions, particularly those that are attachment-theory informed, possess a depth and complexity that makes them difficult to scale with fidelity *[“We’re constantly grappling with the tension of the depth of the clinical model… which is what drew all of us to this model*,* and replicability for scaling. Because scaling implies a breadth of replication*,* right? And it’s hard to maintain both the depth and the breadth. So that’s where we’re at right now of thinking about*,* how do we achieve both without sacrificing the other?” – participant 3]*. Different programs use different methods to support fidelity and quality including strict initial training assessment processes, ongoing data reporting to track fidelity at scaled sites, and Community of Practices as “touch points” to support quality *[“Once folks are out in the world*,* if there’s not that connection back*,* you just don’t know you know what’s happening” – participant 7].* When there are no systems in place to track quality and fidelity, program scalers lack the capacity to understand what is happening at the scaled sites, and to engage in quality improvement *[“what we don’t know is*,* what are the current practices of [intervention]*,* and what’s the quality….this is a big area of interest for us*,* because this needs to inform our work” – participant 15; “One of the challenges we have with this overall is that we can’t monitor and control what’s being delivered out on the ground once it’s out there. So we train people. We offer support to people*,* but it’s up to the people to deliver what we ask them to deliver*,* to deliver with fidelity*,*” – participant 5].*

#### Adaptability and Iteration

Early parenting interventions scale well when there is room in the implementation model for adaptation to meet the needs of different groups and communities. This includes adaptations to the implementation methods *[“For each situation*,* a training type or condition might be better than another” – participant 4]* but also to the program itself *[“They’ve made some tweaks to the model to make it work for them in [location]. But…it’s the same basic model” – participant 6].* Adaptations for different cultural groups enhances consumer acceptability and uptake, and opens new opportunities for impact. Different training and/or support mechanisms may be required, for example, for rural and remote communities, or Indigenous groups. Iterating implementation and treatment models over time can also lead to improved processes and approaches *[“Dissemination of any intervention is about continuous improvement” - participant 14; “…failures led us to make changes…We refined it. We’ve continued to refine that over time. But that’s the process” – participant 16].*

#### Genuine Stakeholder Involvement

Engagement with stakeholders must be genuine and meaningful. Clinicians and agencies feel invested and contribute meaningfully to implementation and broader scale-up efforts when they feel valued as an integral part of a broader team *[“Our goal there is to make them feel valued*,* that we see them as good colleagues here… when we build that kind of relationship with them*,* it keeps their enthusiasm for it*,* and it helps them feel competent” – participant 18*]. It is also important to include stakeholders genuinely in decision-making roles (e.g., forming steering committees or advisory boards comprising a mix of consumers, clinicians, agency leaders, policy-makers and government personnel, with representation from different geographical areas) *[“We gave them something meaningful each time they came….we were trying to make the most out of every minute that we had with them*,* and show them we valued their contributions*,* and show them that it was making a difference for kids and families” – participant 4].* Involving this diverse range of stakeholders in the early stages of the scale-up (e.g., pilot projects) can also increase buy in as the scale-up commences.

#### Community Embedding

For an early parenting program to be successfully scaled, it needs to be embedded into local service systems, and so the implementation model needs to include structures and systems designed to support sustainability and integration. Ideally, over time, external support becomes unnecessary as the model becomes part of standard practice at the local site *[“We actually set it up so that at scale*,* it becomes just embedded usual care. And we disappear out of that” – participant 6].* Sustainability is also enhanced by embedding the program within government policy *[“Thinking through*,* ‘What is the structure that you can build this program into*,* so that it will be sustained?’ Because people come and go*,* we come and go*,* I come and go. Everybody does. But it’s building it into a structure that’s going to be around” – participant 1].*

#### ‘Systems’ Thinking

Successful implementation requires consideration of the place of the program within systems outside it, and there is also a need for cross-system engagement, which takes time, persistence, and funding [*“We need to shift systems so they’re better geared*,* better connected*,* more easily navigable by families” – participant 10].* Work needs to be done to *“leverage existing capabilities and assets”* and *“get creative about using limited resources often that are very siloed” (participant 10)*, which may include delivering new programs within *“existing platforms or service models” (participant 5)* and leveraging existing relationships *[“Finding the people who are committed to it in the State*,* and who are likely to remain and who are in a position to make decisions*,* so that you can keep things moving.” – participant 1].* Thinking more holistically and inclusively about parenting interventions is also recommended, allowing room for multiple interventions to meet the different needs of different families and service providers *[“…figuring out how to get the political buy in*,* get the funding*,* make the pieces work*,* move the workforce*,* hopefully not one person*,* hopefully a bunch of people working together on that. [people thinking about implementation] are having to like*,* map all those pieces and figure out how they fit together and how to sustain them over what are usually short funding cycles*,* uncertain fiscal landscapes” – participant 7].*

### Theme 4: Intervention Quality

Features of the parenting program itself can play an important role in whether it scales well. The most scalable interventions are often ones that are simple, easy to implement, and that resonate with clinicians *[“I think the key to success has been…that clinicians find it understandable. They can transfer their understanding to parents*,* and it sticks with the parents… we’ve trained 70*,*000 people worldwide*,* and if you ask me how it got disseminated*,* I will tell you it got disseminated by word of mouth” – participant 14].* Programs also scale well when they are backed by a strong evidence-base and demonstrated return-on-investment, as this contributes to buy in at all levels *[“The people who are committed to doing this are committed to doing it because it works” – participant 19]*. When evidence-based programs are placed on program ‘menus’ or ‘registries’, there is far greater potential for uptake and scaling.

### Theme 5: Team Composition

#### Champions

Key to any successful scale-up are intervention ‘champions’, or individuals or groups who are passionate about the work and who use their energy and resources to drive the scaling or local implementation efforts forward. *[“It’s having a champion - and that can be a trainer or a funder*,* or an infant mental health association or a hub that helps to coordinate trainings” – participant 3]”.* Program developers and trainers are usually the first key champions [“*I’m enthused about [intervention]. I love it” – participant 18*], but champions in other places are also vital. This includes both champions within policy/government *[“A local champion at a higher level who’s got some funding to support the sustainability” – participant 9]* and local champions who support implementation at the community level *[“At the end of the day*,* in order for an initiative to be successful*,* you have to have that champion - one or two – that are just going to be really excited*,* that’s going to figure things out” – participant 2).* Sometimes champions emerge naturally, but often there is a deliberate identification and cultivation of potential champions, with the purpose of building the *“capacity from within” (participant 13). [“We look for the trainees who exude competence and love for [intervention] and are good at the skills*,* and we invite them to be a trainer*,* and then we invite them to come alongside. And it’s a beautiful way to go*,* and it’s very much relationship based” – participant 18]*

#### Funders

In addition to having people who are passionate advocates for the program and scale-up effort, there also needs to be people involved who have *“the purse strings” (participant 23)* as the ability to acquire and commit funding increase the chances of the intervention being implemented and sustained.

#### Wide Representation of Skills, People, and Stakeholders

Successful scaling efforts are supported by teams of people with the right mix of skills and expertise across multiple domains *[“I had to have the right people in place” – participant 13].* This includes not only clinical and training capabilities but also business acumen, project coordination, marketing, and design. Early decisions about team composition are critical, with strategic partnerships helping to navigate legal, financial, and operational complexities. For example, business professionals may support foundational tasks such as forming corporations and managing documentation, while others may contribute to branding and communication efforts to make academic content accessible to community agencies. The development process often also benefits from collaborations with respected figures in the field such as grant writers and researchers, who add credibility and methodological rigor. Strong coordination and interpersonal skills within the team also play a vital role. Project coordinators who are personable and effective can help unify efforts and maintain momentum.

### Theme 6: Time and Planning

Scaling is a gradual process that rarely follows a uniform or linear path. In most cases, it takes many years to even reach the point where scaling becomes feasible, and progress can remain slow even then. Program developers often begin with small pilot grants and incrementally build the funding, infrastructure, and capacity needed for broader implementation *[“We started small*,* and we started with one small pilot grant*,* and just kept building*,* building and building until we got a large federal grant*,* and then studied the implementation of [intervention] across the state” – participant 4].* In some cases, scaling follows a structured and deliberate trajectory, guided by multidisciplinary planning and implementation science frameworks *[“They were really thoughtful about ‘what implementation framework do we want to use? What are the phases? What do we do?’ And their implementation model is really driven by science too” – participant 13; “It didn’t happen overnight*,* and it didn’t happen by chance.” – participant 8]*. In other cases, however, the scaling process is more organic, with initial training efforts sparking growing demand through word of mouth, and early dissemination occurring without formal planning *[“It was literally not planned on any level*,* except*,* ‘Let’s get something scalable out there that’s built on this platform that we believe in’” – participant 14*]. This can take program developers by surprise *[“they had no idea the demand that would follow*,* and couldn’t keep up with it” – participant 14]*, and these efforts often evolve into effective models through experience and a ‘learning-by-doing’ approach. Regardless of the pathway, successful scale-up typically unfolds over many years and requires sustained commitment, adaptability, and strategic thinking.

## Discussion

This study investigated critical factors that facilitate the successful scale-up of early parenting interventions. Through qualitative analysis, six key factors that enabled successful scaling of early parenting interventions emerged: (1) adopting a business mindset; (2) securing stakeholder buy-in across multiple levels; (3) implementing a robust and flexible implementation approach; (4) ensuring high intervention quality; (5) assembling a team with the right composition of skills and expertise; and (6) time and planning.

One of the major contributions of this study is the emphasis on developing a business ‘model’, with consideration of financial resourcing, commercialisation, and governance. A business model encompasses the strategic process of conceptualising, designing, and operationalising new methods for creating, delivering, and capturing value within an organisation (Wirtz & Daiser, 2017). Given the complexities of the regulatory frameworks, funding structures, and the fragmented and siloed nature of modern health service systems, it is important that business models are not overlooked (Babatunde, 2024; Fieldston et al., 2013). Many participants in the current study spoke of initially feeling unprepared and reluctant to think about their program with a ‘business’ mindset but later realising the necessity of doing so. This is an important and actionable message for developers of early parenting programs, many of whom are clinicians or clinical academics without training or experience in business management. This gap between awareness of the need for business thinking and application in the real world (a phenomena sometimes referred to as the ‘secondary gap’; McNett et al., 2024; Westerlund et al., 2019), can be ameliorated by engaging in formal training/education in business model development, and/or bringing people with the requisite training and experience into the scaling team (see Theme 5).

Securing buy-in across multiple stakeholder levels was also identified as a critical enabler, a finding that aligns with a growing body of implementation science literature emphasising the importance of multi-level stakeholder engagement. The Consolidated Framework for Implementation Research (CFIR; Damschroder et al., 2009), for example, provides a useful lens, highlighting constructs such as leadership engagement, organisational readiness, and external policy alignment as critical to implementation success. Similarly, the Exploration, Preparation, Implementation, Sustainment Framework (EPIS; Moullin & Aarons, 2022) underscores the need for strategic planning and stakeholder involvement across all phases of implementation, particularly in navigating system-level barriers and facilitators. Community engagement strategies, such as those found in Community-Based Participatory Research (CBPR; Wallerstein et al., 2017) further reinforce the value of genuine collaboration with end-users and local organisations to ensure interventions are culturally relevant and contextually appropriate. Together, these frameworks support the conclusion from this study that securing buy-in from stakeholders at multiple levels is not a peripheral task but a central, relational process that underpins sustainable scale-up.

Importantly, this study also provided several practical strategies to foster buy in. For clinicians and agencies, early consultation and relationship-building were essential, with site visits, flexible training formats, and financial incentives fostering engagement and enthusiasm. Buy in from managers and administrators required targeted outreach, with efforts focused on identifying key decision-makers and involving entire teams in training to ensure organisational alignment. Community buy-in was strengthened through genuine collaboration, with developers listening to local needs and adapting interventions to reflect caregivers’ values and preferences. Government engagement was facilitated by aligning interventions with existing policy frameworks, conducting targeted advocacy, and maintaining visibility through regular communication and reporting. One of the strongest take-home message from this study is the importance of achieving integration of early parenting programs into government policy and health service infrastructure – a process that has been described by some as “scaling up” (Moore et al., 2015), and is distinguished from simply ‘spreading’ or replicating an intervention from one location to multiple locations (Bevan et al., 2024).

This study also identified several practical ‘implementation’ factors that contribute to successful scaling, namely: assessing and improving site readiness prior to implementation, building workforce capacity; implementing sustainable processes for clinician training, supervision and fidelity monitoring. These factors correspond to those identified by previous researchers. In one of the most famous models, for example, Fixsen and colleagues (Bertram et al., 2015; Fixsen et al., 2009) reviewed a series of successful implementation programs and identified a core set of implementation components, which they separated into three classes of ‘integrated and compensatory’ implementation drivers: (1) Competency drivers: selection, training, coaching; (2) Organisational drivers: systems level, facilitative administration, decision support and data system; and (3) Leadership drivers: technical, adaptive. It is also important to note the current study’s findings regarding adaptability (having an openness to tailoring the intervention and implementation plans to meet the needs of different groups and situations), embedding (embedding the scaled program into the community), and systems thinking (leveraging/working with existing systems to maximise uptake and sustainability).

The current study also highlights the importance of scaling high quality early parenting interventions. While there was a consensus among participants that proof of quality ideally comes from well-conducted randomised controlled trials, there was also acknowledgement of the importance of other indicators such as the degree to which the intervention was liked and resonated with clinicians, caregivers, and communities. There was a tension for some participants around the competing needs to deliver the intervention as intended and researched, and to adapt and tailor the intervention to meet the cultural needs of specific groups and situations. In keeping with other available viewpoints, there was a sense that interventions models which were more flexible and designed for/open to community adaptation appeared to most scalable.

A final contribution of this study is the reminder of the importance of assembling a team with the right mix of skills to support the scaling effort. Scaling is not a technical or clinical endeavour - it is a complex, multi-dimensional process that requires expertise across domains. Teams must include individuals with clinical knowledge and training capacity, but also those with project coordination skills, business acumen, stakeholder engagement skills, policy awareness, marketing and communications expertise, and design capabilities. These diverse skill sets enable the team to navigate the operational, financial, policy, and relational challenges that arise during scale-up. Scaling early parenting programs is also a values-driven endeavour. Champions for the intervention and more broadly for the scaling effort are required at all levels. Having team members who can engage meaningfully with diverse communities, build trust, and foster genuine collaboration is also essential. Finally, strategic partnerships also emerged as a key enabler, with collaborations between clinicians, academics, policy makers, business professionals, and community leaders helping to bridge gaps in knowledge and capacity. These partnerships not only enhanced operational efficiency but also lent credibility and visibility to the scaling effort. Taken together, these findings suggest that successful scale-up efforts are underpinned by teams that are both technically equipped and relationally attuned. Decisions about team composition should be made with intentionality, ensuring that the team reflects the multifaceted nature of scaling and can respond to the evolving demands of implementation in diverse contexts.

This study offers valuable insights into the factors that facilitate the successful scale-up of early parenting interventions; however, several limitations should be acknowledged. First, the qualitative design, while rich in depth and context, inherently limits the generalisability of findings. The perspectives captured reflect the experiences of a specific group of experts and may not represent the full diversity of stakeholders involved in scaling efforts globally. In particular, the majority of participants were from higher income countries, and it is possible that their views on scaling may not be applicable to low- and middle-income countries. Second, the sample size, though adequate for qualitative analysis, remains relatively small and geographically concentrated, with limited representation across different countries. This restricts the applicability of findings to more resource-constrained settings, and raises questions about how culture and local system differences may influence scaling. Limited demographic information about participants further restricts understanding of the sample. Third, participant selection may have introduced bias, as individuals were recruited based on their known involvement in scaling initiatives. This likely skewed the data toward more successful or well-resourced efforts, overlooking valuable lessons from less visible or unsuccessful attempts. Fourth, the reliance on retrospective self-reporting introduces the possibility of recall bias and social desirability bias, as participants may unintentionally emphasise positive outcomes or minimise challenges. Finally, there was an over-representation of participants speaking about the PCIT intervention and it is possible that different themes may have emerged had a different selection of programs been included. While these limitations are important to recognise, the findings nevertheless represent useful considerations for scaling endeavours.

### Conclusion

The study is important because it builds on evidence of the effectiveness of early parenting interventions to explore, for the first time, the processes through which early parenting interventions can be implemented in the real world, at scale. The study identified six key factors that support the successful scale-up of early parenting interventions: adopting a business mindset, securing multi-level stakeholder buy-in, applying a flexible implementation model with a focus on supporting quality and sustainability, choosing a high-quality intervention, building a multidisciplinary team, and recognising that scaling may follow either planned or organic pathways. A consistent theme across the data was that successful scale-up requires time, collaboration and dedication, iterative learning, and responsiveness to local contexts. Flexibility in delivery models, openness to adaptation, and a systems-thinking approach are essential to navigate the complexities of different environments. Further investigation is needed into how different scaling strategies perform across contexts, particularly in specific populations such as specific cultural groups or rural communities.

## Data Availability

Data will be available from the study authors upon reasonable request.
